# Bacterial clonal diagnostics as a tool for evidence-based empiric antibiotic selection

**DOI:** 10.1371/journal.pone.0174132

**Published:** 2017-03-28

**Authors:** Veronika Tchesnokova, Hovhannes Avagyan, Elena Rechkina, Diana Chan, Mariya Muradova, Helen Ghirmai Haile, Matthew Radey, Scott Weissman, Kim Riddell, Delia Scholes, James R. Johnson, Evgeni V. Sokurenko

**Affiliations:** 1 Department of Microbiology, University of Washington School of Medicine, Seattle, WA, United States of America; 2 Kaiser Permanente Washington, Seattle, WA, United States of America; 3 ID Genomics, Inc., Seattle, WA, United States of America; 4 Children’s Hospital, Seattle, WA, United States of America; 5 Kaiser Permanente Washington Health Research Institute, Seattle, WA, United States of America; 6 VA Medical Center and University of Minnesota, Minneapolis, Minnesota, United States of America; Leibniz-Institute DSMZ, GERMANY

## Abstract

Despite the known clonal distribution of antibiotic resistance in many bacteria, empiric (pre-culture) antibiotic selection still relies heavily on species-level cumulative antibiograms, resulting in overuse of broad-spectrum agents and excessive antibiotic/pathogen mismatch. Urinary tract infections (UTIs), which account for a large share of antibiotic use, are caused predominantly by *Escherichia coli*, a highly clonal pathogen. In an observational clinical cohort study of urgent care patients with suspected UTI, we assessed the potential for *E*. *coli* clonal-level antibiograms to improve empiric antibiotic selection. A novel PCR-based clonotyping assay was applied to fresh urine samples to rapidly detect *E*. *coli* and the urine strain's clonotype. Based on a database of clonotype-specific antibiograms, the acceptability of various antibiotics for empiric therapy was inferred using a 20%, 10%, and 30% allowed resistance threshold. The test's performance characteristics and possible effects on prescribing were assessed. The rapid test identified *E*. *coli* clonotypes directly in patients’ urine within 25–35 minutes, with high specificity and sensitivity compared to culture. Antibiotic selection based on a clonotype-specific antibiogram could reduce the relative likelihood of antibiotic/pathogen mismatch by ≥ 60%. Compared to observed prescribing patterns, clonal diagnostics-guided antibiotic selection could safely double the use of trimethoprim/sulfamethoxazole and minimize fluoroquinolone use. In summary, a rapid clonotyping test showed promise for improving empiric antibiotic prescribing for *E*. *coli* UTI, including reversing preferential use of fluoroquinolones over trimethoprim/sulfamethoxazole. The clonal diagnostics approach merges epidemiologic surveillance, antimicrobial stewardship, and molecular diagnostics to bring evidence-based medicine directly to the point of care.

## Introduction

The rising prevalence of antimicrobial resistance among bacterial pathogens is one of today's greatest medical challenges [1–4]. Because conventional cultures can take days to determine a pathogen's antimicrobial susceptibility profile, new approaches are needed urgently to guide empiric (pre-culture) selection of antimicrobial therapy.

Urinary tract infection (UTI), a leading reason for antibiotic treatment among women and elderly men, is caused mainly by *Escherichia coli* [5–7]. Patients with UTI symptoms account for a large share of visits to emergency departments and urgent care clinics [8]. Correct empiric UTI therapy is essential for prompt symptomatic relief and to prevent progression and relapse [9, 10].

Empiric antibiotic selection for UTI is guided by the syndrome (e.g., uncomplicated cystitis vs. pyelonephritis) and the local species-level resistance prevalence among uropathogens. As a first choice for empiric treatment of uncomplicated UTI (i.e., cystitis), the Infectious Diseases Society of America (IDSA) recommends trimethoprim-sulfamethoxazole (T/S) if the local T/S resistance prevalence in *E*. *coli* is ≤ 20%, whereas for pyelonephritis, empiric fluoroquinolone (FQ) therapy is suggested if the local FQ resistance prevalence in *E*. *coli* is ≤ 10% [11].

Due to increasingly prevalent resistance, these resistance thresholds are becoming difficult to implement. In many centers, the prevalence of T/S resistance in *E*. *coli* considerably exceeds 20%, leading to greatly diminished empiric T/S use [12–14]. Use of the IDSA-recommended next-choice antibiotics for uncomplicated UTI–nitrofurantoin (NIT) and fosfomycin (FOS)–remains uncommon, despite a low prevalence of resistance. In contrast, use of FQs for uncomplicated UTI has surged, despite recommendations to reserve FQs for more challenging infections [11, 15–18]. Unfortunately, the prevalence of FQ resistance in *E*. *coli* now well exceeds 10% (or even 20%) in most locales, complicating guideline-adherent use of FQs too.

Uropathogenic *E*. *coli* can be split into multiple clonal groups that differ from each other and the species overall in the prevalence of resistance to specific antibiotics [19–21]. Clonal resistance profiles are sufficiently stable across time periods, geographic regions, and patient populations to allow their use to guide empiric treatment selection [22–24].

Here, we assessed how a clonal diagnostics approach might improve point-of-care empiric antibiotic selection. Specifically, within a prospective observational cohort, we implemented a novel quantitative PCR (qPCR)-based test that resolves over 50 *E*. *coli* clonal groups. We found that the clonal diagnostics approach could significantly increase the use of T/S and other agents over FQs, while significantly reducing the risk of antibiotic/pathogen mismatch.

## Materials and methods

### Local reference set

The local reference *E*. *coli* isolates (n = 1,225, S1 Dataset) were obtained in sequential batches, without pre-selection, from urine samples submitted to the clinical laboratory at Kaiser Permanente Washington (KPWA: Seattle, WA) between November, 2010 and April, 2014. Antibiotic susceptibility profiles were determined by disk diffusion according to CLSI guidelines [25]. Here, we focused on susceptibility to seven antibiotics–ampicillin (AMP), cefazolin (CZ), ceftriaxone (CTR), T/S, FQ, NIT, and FOS. The reference isolates' clonal identity was determined by a recently described novel 7-SNP test [19], which assigns isolates to over 50 clonotypes–clonal groups corresponding with multi-locus sequence types (STs) or combinations thereof (S1 Table).

Each clonotype was assessed for the corresponding isolates' cumulative antibiogram to each of the above 7 antibiotics (S1 Table).

### Multi-national reference set

The multi-national reference set included 741 clonotyped non-Seattle *E*. *coli* isolates from a previously described multi-center collection, with their clonotype-specific antibiograms [19] (S2 Dataset). These non-pre-selected isolates had been collected between October 2010 and June 2013 from 4 clinical laboratories in Minneapolis, Minnesota (Veterans Affairs Medical Center, 109 isolates); Muenster, Germany (University Hospital, 386 isolates); Moscow, Russia (Gemotest Center, 53 isolates); and Wrocław, Poland (Medical University of Wrocław, 193 isolates).

### Prospective clonal diagnostics study

The study was done from July 2014 to November 2015 at the KPWA Urgent Care Clinic (Capitol Hill, Seattle), assisted by the KPWA central laboratory and the Department of Microbiology, University of Washington (UW; Sokurenko laboratory). Study participants were patients from 18 to 90 years old (mean 52.5 years, ± 22.8; females 82%), who presented with symptoms of UTI. The Institutional Review Board (IRB) granted a waiver of consent for collection and use of the samples. Per the clinic's standard protocol, urine specimens submitted to the Urgent Care Clinic laboratory underwent urinalysis using the Bayer Multistix strip, with results (determined per the manufacturer's instructions) reported within 3 min of testing.

Because this urinalysis test identifies > 98% of E. coli -positive urine samples (not shown), the 7-SNP test was performed only on urinalysis-positive specimens.

All the data were analyzed anonymously (identifiers were removed in the Kaiser Permanente Washington Urgent Care clinical lab). De-identified electronic medical records were reviewed according to an Institutional Review Board-approved protocol to identify antibiotics prescribed on the day of the index visit or the following day.

### 7-SNP test

The qPCR-based 7-SNP test was performed essentially as described previously [19], with minor modification (see below). The test determines the clonotype identity of the bacteria based on a combinatory number (barcode) derived from the presence/absence of seven single nucleotide polymorphism (SNP) positions within two genes, *fumC* and *fimH*. An additional test reaction determines presence of the *uidA* locus, which is specific to *E*. *coli*. The qPCR test was performed using the Rotor-Gene Q instrumentation platform (QIAGEN, Inc). The Rotor-Gene qPCR tube strips, functionalized with reaction mixes containing SNP- and *uidA*-specific primers, as described previously [19], were supplied by ID Genomics, Inc. (Seattle, WA) and stored at -20°C until use. To shorten the test time, two minor modifications were made to the previously described protocol. First, for bacterial lysis and crude DNA extraction, the bacteria/Chelex beads pellet was heated at 96°C for 3 minutes instead of 5 minutes as described previously. Second, the bacterial DNA was added to the functionalized qPCR test tubes *after* the latter were pre-heated for 3 min as a ‘jump start’ step (that was done in parallel to the bacterial DNA prep) and not *before* the pre-heating step as described previously. Neither step affected the test quality.

### Urine culture and isolate testing

Each urine specimen also underwent quantitative culture, species identification and susceptibility testing according to standard procedures. A clinically significant bacterial load was defined as ≥ 10^3^ cfu/ml [26].

### Statistical analysis

Statistical analysis was performed using STATA 14.0 (StataCorp LP, USA). Test performance characteristics estimates and confidence intervals were calculated using a logistic model with a positive test as the predictor and a positive culture as the outcome. Correlation between time to the positive test and *E*. *coli* load as detected by culture was analyzed using linear regression, with bacterial load given as log_10_ cfu/ml. Comparisons between the study and reference isolate sets for the prevalence of the major constituent clonotypes were done using a chi-square test of independence or Fisher’s exact test (if required). Resistance prevalence for all *E*. *coli* isolates within the study and reference sets was compared individually for each antibiotic using a two-sided Fisher’s exact test. The difference in overall resistance prevalence between study and reference isolates was additionally evaluated using multivariable logistic regression, unadjusted or adjusted for the major clonotype CT561. Disparities between resistance of major clonotypes within study set and either of the two reference sets in clonotype/antibiotic profiles for major clonotypes were compared using McNemar test.

### Calculations of prescription mismatch rates

Prescription mismatch rates were calculated for a subset of cases where *E*. *coli* was detected both in the 7-SNP test and by culturing, and an antibiotic was prescribed at the index day of the specimen collection. *Observed* prescription mismatch rate was calculated for individual classes of antibiotics and overall as the percent of cases where *E*. *coli* isolates were resistant to the prescribed antibiotic according to the isolates' culture-based antibiogram. *Expected* mismatch rate was calculated for individual classes of prescribed antibiotic based on the rate of prescription of this antibiotic in the study patients and the overall rate of resistance to that antibiotic in the study *E*. *coli* isolates according to the culture-based antibiograms. *Predicted* mismatch rate was calculated for individual classes of prescribed antibiotic as follows: in each case the drug was considered as an acceptable choice (“allowed”) for a strain of a particular clonotype if in the reference set the resistance prevalence in that *E*. *coli* clonotype was not above the threshold level of 20% [11]. Otherwise, use of that antibiotic would be rejected (“disallowed”). If an antibiotic was allowed for a particular isolate, but this isolate happened to be resistant to this drug according to its culture antibiogram, it was defined as a *predicted* mismatch. Mismatch rates were compared using Fisher’s exact test.

## Results

### 7-SNP test determination of *E*. *coli* presence and load

Of 750 urinalysis-positive samples from urgent care patients with suspected UTI, 274 (36%) contained *E*. *coli* according to the 7-SNP test (Table 1, S3 Dataset). Quantitative culture detected *E*. *coli* at ≥ 10^2^ cfu/ml (the detection limit) in 267 (97%) 7-SNP-positive and 39 (8%) 7-SNP-negative specimens, and at ≥ 10^3^ cfu/ml (the clinical significance threshold) in 257 (94%) 7-SNP-positive and 2 (0.4%) 7-SNP-negative specimens. Thus, for detection of *E*. *coli*, for samples with ≥ 10^2^ cfu/mL the 7-SNP test's performance characteristics were sensitivity 87.7%, specificity 98.4%, positive predictive value 97.5%, and negative predictive value 91.8%, whereas for samples with ≥ 10^3^ cfu/ml they were 98.4%, 96.5%, 93.8%, and 99.6%, respectively (Table 1).

**Table 1:** Performance characteristics of the 7-SNP test for detecting *Escherichia coli* in urine samples.

	Standardized culture result	
7-SNP test result or performance characteristic	Any *E*. *coli* (N = 306)	≥ 10^3^ cfu/ml *E*. *coli* (N = 259)
**Positive (N = 274)**	267	257
**Negative (N = 476)**	39	2
**Sensitivity** ^a^**, %**	87.3 (83.0–90.8)	99.2 (97.2–99.9)
**Specificity** ^a^**, %**	98.4 (96.8–99.4)	96.5 (94.5–98.0)
**PPV** ^a^**, %**	97.5 (94.8–99.0)	93.8 (90.3–96.3)
**NPV** ^a^**, %**	91.8 (89.0–94.1)	99.6 (98.5–99.9)

^a^ 95% confidence intervals are given in parenthesis after point estimate.

In 7-SNP negative samples neither *E*. *coli*-specific *uidA* nor any SNP-specific primers gave a distinct positive signal, indicating the inability to detect *E*. *coli* in those samples is not likely to be due to the lack of 7-SNP markers in the strains. The 7-SNP test’s failure to detect *E*. *coli* in 37 of 47 specimen with a bacterial load <10^3^ cfu/ml likely indicates the limit of qPCR-based detection of *E*. *coli* under the current protocol. The 7-SNP test’s failure to detect *E*. *coli* in 2 of 259 specimen with a bacterial load ≥ 10^3^ cfu/ml could also indicate the presence of qPCR inhibitors in the urine sample.

Of 306 urine samples where *E*. *coli* was cultured, 45 (15%) had other microorganisms cultured as well. In 32 (71%) of the 45 polymicrobial cases, the *E*. *coli* load was <10^3^ cfu/ml, and only 6 of yielded a positive 7-SNP test. In contrast, in the remaining 13 mixed cases, which had an *E*. *coli* load ≥ 10^3^ cfu/ml, the 7-SNP test was uniformly positive. Among the 19 polymicrobial cases with a positive 7-SNP test, the majority (90%) contained enteroccoci or other Gram-positive species as the non-*E*.*coli* bacteria, and *E*. *coli* was a minority species in only 3 cases, i.e., <1% of all 306 cases. In 7 of 19 polymicrobial cases *E*. *coli* was by far the most prevalent organism. For further analysis, presence of non-*E coli* species in polymicrobial specimens was disregarded.

On average, the 7-SNP test detected *E*. *coli* in urine specimens in 20 qPCR cycles, or 20.5 ± 3.4 min. Time to detection corresponded inversely with *E*. *coli* load (S1 Fig). Including the 8-min sample preparation time, the 7-SNP test could detect on average a clinically significant *E*. *coli* load in 28.0 ± 3.4 minutes.

### Clonotypes in urine samples vs. cultured isolates

**Fig 1:**
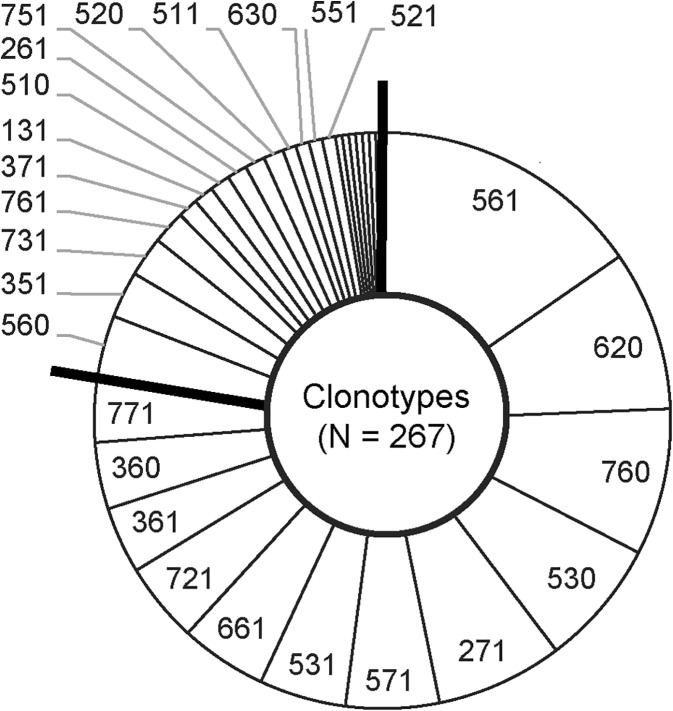
Clonotypes identified in study urine samples by the 7-SNP test. Solid lines separate the 12 most frequent clonotypes (≥ 12 samples each) from the minor clonotypes.

In urine samples that contained *E*. *coli* according to the 7-SNP test, clonotype identity could be defined in 22 PCR cycles (or 23.0 ± 3.2 minute), or 31.0 ± 3.2 minutes after sample availability. Among the 267 samples that contained *E*. *coli* by both the 7-SNP test and culture, in 260 (97%) the clonotype determined directly in urine corresponded with that determined by single-colony testing. Of the seven discrepant samples, four had < 10^3^ cfu/ml and one had two clonotypes by single colony testing, one of which matched the 7-SNP test-determined clonotype. The 268 culture-confirmed *E*. *coli* study isolates represented 33 clonotypes (Fig 1), 31 of which were present in the local reference set (S1 Table), with the two novel clonotypes represented by a single isolate each.

### Overall vs. clonal antibiograms

**Fig 2:**
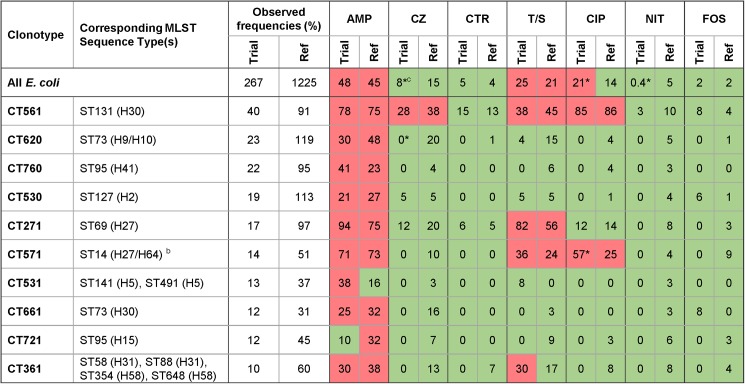
Comparison of antibiotic resistance prevalence among study vs. local reference *Escherichia coli* isolates for the 10 most frequent study clonotypes. ^a^ For each CT (clonotype) the most prevalent (≥ 90% of isolates) sequence type (ST), as determined by multilocus sequence typing, is shown, with its subclone determined by CH typing (H) in parentheses, where applicable. For CTs represented by multiple STs (each accounting for ≤ 90% of the constituent isolates) up to four major STs and the corresponding H subclones are listed. ^b^ CT571 comprises ST14 (H27), ST404 (H27) and ST1193 (H64), which all belong to clonal complex of ST14. ^c^ For the study set and local reference set (‘Ref’), antibiotic resistance prevalence for all *E*. *coli* and for major clonotypes is given as percent of resistant isolates in relation to the total number of isolates, overall or within the clonotype; green color indicates resistance below conventional threshold (≤ 20%), red color–above it (> 20%); the resistance within study and reference sets was compared individually for each antibiotic using a two-sided Fisher’s exact test; * P < .05.

Study and local reference isolates had a similar overall prevalence of resistance to AMP (48% vs. 45%), T/S (25% vs. 21%), CTR (5% vs. 4%), and FOS (2% for both) (Fig 2). The study isolates had a higher prevalence of resistance to FQs (21% vs. 15%, P *=* .004) and a lower prevalence of resistance to CZ (8 vs. 15%, P *=* .001) and NIT (0.4 vs. 5%, P *<* .001).

Among the study isolates, 12 clonotypes accounted for ≥ 10 isolates each, and for 78% of the collection (Fig 1). The only significant clonotype-specific resistance prevalence differences between the study and reference isolates involved CZ in CT620 (0% vs. 20%, respectively) and FQs in CT571 (57% vs. 25%, respectively) (Fig 2). Likewise, at a 20% resistance threshold for allowed empiric use of an antibiotic, the local reference set disagreed with the study set for only 3 antibiotic-clonotype combinations, i.e., AMP in CT531 and CT721, and T/S in CT361 (Fig 2). The observed difference between the study and reference database with respect to the resistance profiles of a few clonotypes could be due at least in part to a possible differential prevalence of sub-CT clonal groups that are not recognized as separate clonotypes by the current resolution of the 7-SNP test.

Notably, the difference in overall FQ resistance prevalence between the reference and study isolates became non-significant (P = .51) after adjusting for the corresponding difference in prevalence of CT561 (7.4% vs. 16.3%, respectively: OR 2.42, CI 95% 1.66–3.59, *P <* .001), which corresponds with the pandemic multi-drug resistant clonal group ST131-H30. Thus, differential inter-clonal rather than intra-clonal variability in resistance prevalence is a critical determinant of overall resistance patterns.

### Antibiotic prescribing and mismatches for study subjects

An antimicrobial prescription accompanied the index visit for 220 (82%) of 267 subjects with urinary *E*. *coli* according to both the 7-SNP test and culture. Most prescriptions (116, 53%) were for FQs, followed by T/S (63 29%), CZ (19, 9%), and NIT (100, 5%) (Table 2). Other antibiotics included the AMP congener amoxicillin (3 prescriptions), CTR (2), 2^nd^-generation cephalosporins (3), amoxicillin/clavulanate (2), clindamycin (1), and vancomycin (1).

**Table 2:** Prescription of antibiotics.

Prescribed antibiotics ^a^	No. prescriptions (% of Total)	Antibiotic-pathogen mismatch (% of no. prescriptions)	Prescription allowed by 7-SNP test (% from Total) ^b^	Antibiotic-pathogen mismatch after 7-SNP test ^c^	
				Allowed cases (%)	Disallowed cases (%)
**FQ**	116 (52.7)	16 (13.8)	173 (78.6)	8 (4.6)	36 (77)
**T/S**	63 (28.6)	14 (22.2)	126 (57.3)	10 (7.9)	44 (47)
**CZ**	19 (8.6)	2 (10.5)	162 (73.4)	7 (4.3)	8 (14)
**NIT**	10 (4.5)	0 (0)	220 (100)	1 (0.5)	N/A
**AMP**	3 (1.4)	2 (66.7)	18 (8.2)	6 (33.3)	106 (53)
**CTR**	2 (0.9)	0 (0.0)	220 (100)	9 (4.1)	N/A ^d^
**Other**	7 (3.2)	4 (57)	NC	NC	NC ^d^
**TOTAL (N = 220)**	220 (100)	38 (17.3)	N/A	N/A	N/A

^a^ Abbreviations shown are for the antibiotics to which susceptibility was tested, and are used here to represent the different classes of antibiotics that were prescribed. The actual antibiotics prescribed, by class, were (no. of cases): “FQ” ciprofloxacin (112) and levofloxacin (4); “CZ” cephalexin (19); “AMP” amoxicillin (3); “CTR” cefixime (2); “other” cefuroxime (3), amoxicillin/clavulanate (2), clindamycin (1), and vancomycin (1).

^b^ Among these 220 samples, in what number of samples the clonotype identified in 7-SNP test would have the average resistance level less than cutoff (20% in this instance) in the reference set of *E*. *coli* (1,225 Kaiser Permanente Washington urine isolates in this instance). N/A–not applicable; NC–not considered in the study.

^c^ Among the samples for which the use of this antibiotic was allowed or rejected, how many contained *E*. *coli* isolate resistant to this antibiotic; for Total number of samples the cumulative mistake was calculated using mistakes for each individual class of antibiotics weighted by its prescription rate.

^d^ N/A–not applicable; NC–not considered in the study.

Overall, for 38 of 220 subjects (17.3%) the infecting strain was resistant to the prescribed antibiotic, resulting in antibiotic/pathogen mismatch (Table 2). The observed mismatch rate was not different (P = .39) from the mismatch rate expected from specific resistance prevalence in the study set (see Materials and Methods), suggesting that the prescribers had no special insight into the probability of resistance.

### Antimicrobial therapy options allowed by the 7-SNP test

We next evaluated how effectively the 7-SNP test could guide empiric therapy if used in conjunction with local reference isolate clonal antibiograms and a 20% allowed resistance threshold for empiric use. (For the 2 novel and 4 unidentified CTs, population-wide *E*. *coli* resistance values were used.)

Among the 220 *E*. *coli -*positive study subjects who received antibiotics, the 7-SNP test would allow use of FQs for 78.6%, T/S for 57.3%, CZ for 73.6%, and NIT for 100% (Table 2), with a resulting frequency of antibiotic/pathogen mismatch of only 4.6%, 7.9%, 4.3%, and 0.3%, respectively. Additionally, it would allow use of CTR for 100% (4.1% mismatch), FOS for 100% (1.9% mismatch), and AMP for 8.2% (33% mismatch).

In contrast, if prescribers were to prescribe drugs disallowed by the 7-SNP test, antibiotic-pathogen mismatch would be much more frequent, i.e., 77% for FQs (P < .001), 47% for T/S (P < .001), and 14% for CZ (P = .014) (Table 2). Importantly, for the actual antibiotic/pathogen mismatches observed in the study, the 7-SNP test would have disallowed the prescribed drug in 15 (94%) of 16 instances involving FQs and 11 (79%) of 14 involving T/S.

### Alternative antimicrobials allowed by the 7-SNP test

As noted above, the 7-SNP test would have allowed NIT for all 220 *E*. *coli* -positive, antibiotic-treated patients and at least one of the other three most frequently used antibiotics (FQs, T/S, and CZ) in 173 cases. In these 173 cases, all three of these non-NIT agents would have been allowed for 103 patients (46.8%); FQs and CZ, but not T/S, for 45 (20.5%), and FQs and T/S, but not CZ, for 23 (10.5%) (Table 3). Thus, in addition to NIT, both T/S and FQs would have been allowed in 126 of 173 (72.8%) cases, and both CZ and FQs in 148 of 173 (86.8%) cases, whereas FQs alone (but not T/S or CZ) would have been allowed in just 2 (0.9%) cases.

**Table 3:** Distribution of cases when 7-SNP test allowed the use of FQ ^a^, T/S ^a^, and/or CZ ^a^.

Allowed antibiotic ^a^			No. of cases (% of 220)
FQ ^a^	T/S ^a^	CZ ^a^	
YES	YES	YES	103 (47%)
YES	YES	NO	23 (11%)
YES	NO	YES	45 (21%)
YES	NO	NO	2 (1%)
NO	NO	YES	14 (6%)
Total allowed for FQ, T/S, or/and CZ			187 (85%)

^a^ FQ, fluorquinolones, T/S, trimethoprim-sulfamethoxazole, CZ, 1^st^ generation cephalosporins

One can estimate that if FQs, T/S, and CZ were used in accordance with the 7-SNP test, but in the observed proportions (Table 2), the cumulative antibiotic/pathogen mismatch rate would be 8.0%, as compared with the observed mismatch rate of 19.1% (Table 4). If instead T/S were to be used preferentially when allowed by the 7-SNP test (consistent with IDSA guidelines), followed by FQs or CZ as the 2^nd^ or 3^rd^ choice only if T/S was rejected, the combined mismatch rate would be 7.5%-9.1%. Thus, use of the 7-SNP test to guide empiric antibiotic selection could allow significantly increased T/S use and decreased FQ use, while significantly reducing the risk of antibiotic/pathogen mismatch.

**Table 4:** Prescription rate and antibiotic-pathogen mismatch in different scenarios of antibiotic choice.

Frequency of use of the 1^st^, 2^nd^ and 3^rd^-choice antibiotics (prescription rate, %) ^a^			Total antibiotic-pathogen mismatch rate ^b^		P value
FQ ^a^	T/S ^a^	CZ ^a^	Predicted	Expected	
Observed^c^ (53)	Observed^c^ (29)	Observed^c^ (9)	8.0	19.1	.001
2^nd^ (25)	1^st^ (67)	3^rd^ (8)	9.1	22.7	< .001
3^rd^ (1)	1^st^ (67)	2^nd^ (32)	7.5	19.6	< .001
1^st^ (93)	2^nd^ (0)	3^rd^ (8)	4.8	20.0	< .001
1^st^ (93)	3^rd^ (0)	2^nd^ (8)	4.8	20.0	< .001
3^rd^ (1)	2^nd^ (12)	1^st^ (87)	4.3	10.2	.022
2^nd^ (12)	3^rd^ (0)	1^st^ (87)	3.7	9.7	.018

^a^ Present analysis includes only 187 cases where at least one of the top three groups of antibiotics can be recommended based on the results of the 7-SNP test. Drug abbreviations: FQ, fluorquinolones; T/S, trimethoprim/sulfamethoxazole; CZ, 1^st^-generation cephalosporins.

^b^ Mismatch rate is calculated either based on the resistance prevalence among the isolates when the use of antibiotic was allowed by 7-SNP test (*Predicted*), or based on overall antibiotic resistance prevalence among the study isolates (*Expected*). P value was calculated using two-sided Fisher’s exact test

^c ‘^Observed’ indicates the prescription rate for these three classes of antibiotics that was observed in the study patients (see Table 2).

### Effect of using different resistance thresholds

We next estimated how the use of different arbitrary resistance thresholds (10% or 30%, instead of 20%) would affect allowed antibiotic use, with a focus on T/S. With a 10% threshold, T/S would be allowed in only 37% of cases (vs. 57% with the 20% threshold; P < .001), but with only 3.7% resulting mismatches (vs. 7.9% with the 20% threshold, P = .22; S2A Fig). With a 30% threshold, T/S would be allowed in 71% of cases (vs. 57%, P *=* .004), with 15% resulting mismatches (vs. 7.9%, P *=* .074), which is still lower than the IDSA-suggested 20% resistance prevalence threshold for cystitis, and the observed 25% mismatch rate. Notably, with a 30% resistance threshold, T/S would be allowed as an alternative to FQs in fully 82% of cases (vs. only 73% with the 20% threshold, P *=* .055).

### Effect of using multi-national vs. local reference databases

We estimated next the effect of using clonotype-specific antibiograms from a multi-national (non-Seattle) reference set, rather than the local reference set, to determine clonotype-guided use of antibiotics. Clonotype/antibiotic resistance prevalence profiles for the top 10 study clonotypes were compared head-to-head between the study set and both reference sets, which were compiled, respectively from the local and multi-national isolate collections (S2 Table).

As noted above for the local reference database, with the multi-national reference database the major clonotypes usually did not differ significantly from the study clonotypes for the prevalence of resistance to the six antibiotics tested–AMP, CZ, CTR, FQ, T/S and NIT (resistance to fosfomycin was not determined in the multi-national dataset). However, significant resistance prevalence disparities vis-a-vis the study isolates were somewhat more frequent with the multi-national reference isolates (i.e., 7/60 clonotype/antibiotic combinations) than with the local reference isolates (2/60 total clonotype/antibiotic combinations) (P = .059 in McNemar’s test). The most pronounced difference between the multi-national and local reference databases for non-concordance with the study isolates was when a 10% resistance threshold was used to classify clonotypes as susceptible vs. resistant to a given antibiotic. With this threshold, whereas the local reference clonotypes were non-concordant with the study clonotypes in 5 clonotype/antibiotic combinations, the multi-national reference clonotypes were non-concordant in 19 combinations (P < .001). In contrast, with a 20% resistance threshold, the local and multi-national reference clonotypes did not differ significantly for non-concordance with the study clonotypes (3 vs. 7 clonotype/antibiotic combinations, respectively, P = .16). With a 30% threshold even less non-concordance was observed (9 vs. 12 combinations, respectively, P = .26).

Although the multi-national reference database was not quite as accurate as the local reference database for predicting resistance among the study isolates, with the IDSA-recommended 20% resistance threshold its use would reduce only slightly the frequency of allowed T/S use relative to use of the local reference– 51% vs. 57%, respectively (P = .11) (S2 Fig). Also, at this threshold, use of the multi-national vs. local reference would raise insignificantly the associated antibiotic/pathogen mismatch rate in T/S– 8.9% vs. 7.9% (P = .48). At the alternative resistance thresholds (10%, 30%), however, T/S use would be allowed for fewer cases based on the multi-national reference database than the local reference database– 22% vs. 37%, respectively, for the 10% threshold (P < .001), and 57% vs. 71%, respectively, for the 30% threshold (P = .002) (S2 Fig). Still, the resulting mismatch rate would not differ significantly– 7.1% vs. 3.7%, respectively, for the 10% threshold (P = .40) and 10.4% vs. 14.8%, respectively, for the 30% threshold (P = .14).

Thus, a reference clonotype database that does not include local data, despite not being as concordant with the study isolates as the local clonotype database, could still be used for clonotype-based diagnostics and yield a significant reduction in mismatch rates with empirical antibiotic use.

## Discussion

In this prospective observational cohort study we assessed a prototypic clonal diagnostics method for guiding selection of empirical therapy for *E*. *coli* UTI. Our findings support three main conclusions. First, when the test is performed at the point of care (here, a busy metropolitan urgent care clinic), it can detect *E*. *coli* and determine clonal identity with high specificity and sensitivity within 25–35 min of urine specimen availability, which is an acceptable timeframe for empiric antibiotic prescription. Second, if empiric prescribing were guided by clonal antibiograms from a pre-existing reference database, the frequency of antibiotic/pathogen mismatch could be reduced considerably. Third, use of clonal diagnostics could promote antimicrobial stewardship by encouraging empiric use of preferred antibiotics (e.g., T/S) over less preferred antibiotics (e.g., FQs).

Clonotype-level antibiograms provide much more accurate guidance for empirical treatment than do species-level antibiograms, the current standard [11, 19, 20]. In our population, adherence to IDSA-recommended resistance thresholds would preclude T/S for empiric therapy of uncomplicated cystitis due to the 25% resistance prevalence (cf. the suggested 20% threshold). In contrast, according to clonal antibiograms, T/S conceivably could be used empirically for twice as many patients as actually observed (i.e., from 29% to 57%), yet with an almost 67% relative reduction of the frequency of antibiotic/pathogen mismatch (from 22% to 8%). Similarly, given the ~20% overall prevalence of FQ resistance, empiric FQ therapy was marginally acceptable even for uncomplicated cystitis, and was definitely unacceptable for pyelonephritis. In contrast, if guided by clonal diagnostics, empiric FQ therapy could be used for almost 80% of isolates, with only a 4.6% mismatch, which would qualify well for empiric treatment even of pyelonephritis.

As had been reported previously, the reagent cost of the clonotyping test is already quite low (< $4), and the hands-on time could be reduced significantly with future test optimization. We expect that the main cost-benefit advantage of using clonal diagnostics would be in reducing the prescription of ineffective antibiotics, while constraining overuse of broad-spectrum and/or last-lane antimicrobials. Indeed, while the commonly used antibiotics are relatively cheap (but vary significantly in frequency of use in relation to provider), prescription of an antibiotic regimen to which the *E*. *coli* isolate is resistant is strongly associated with clinical persistence and treatment failure and, thus, need for new prescriptions, extended patient discomfort, repeated outpatient visits or prolonged hospital stay [27–29]. However, quantifying the cost benefits of the improved accuracy of antibiotic prescription allowed by the 7-SNP test would require a separate study.

Although patient-specific factors (e.g., drug allergy history, renal dysfunction, pregnancy, and drug-drug interactions) often constrain prescribers' antibiotic choices, clonal diagnostics could reduce empiric FQ overuse by allowing safe substitution of T/S in nearly 3 of 4 cases when both drugs are allowed. Additionally, with clonal diagnostics guidance, expanded use of 1^st^ generation cephalosporins could further diminish empiric FQ use, which could be especially useful in children and pregnant women, in whom FQs (and sometimes T/S) may be undesirable.

Rapid molecular tools have been explored for antimicrobial resistance prediction by targeting genetic resistance markers [30, 31]. Unfortunately, resistance to a given drug often depends on the presence and proper expression of a wide range of genes and variants. In *E*. *coli* alone, at least 14 different genes can confer T/S resistance, and a dozen different mutations in three chromosomal genes as well as from any of multiple plasmid-borne genes can confer FQ resistance [32–35]. Thus, accurate prediction of resistance/susceptibility to a broad panel of antibiotics currently remains infeasible for a test based only on resistance gene markers.

Before broader clinical implementation, the studied clonotyping test would benefit from technological improvements, including a) fewer hands-on steps and automation of the read-out; b) increased sensitivity to detect low concentrations of *E*. *coli* that still could be indicative of UTI; c) finer clonotyping resolution to identify distinct sub-CT clonal groups, and, potentially, d) use of cheaper and point-of-care-friendly non-PCR DNA amplification/detection platforms. Nonetheless, the current findings provide solid proof-of-principle that a clonal diagnostic approach can optimize selection of empiric therapy for *E*. *coli* UTI. Due to their temporal and geographic stability, clonal antibiograms also supersede species-level antibiograms in being usable in different locales and over several years [22–24]. In the future, clonal reference databases could also include data on a broad range of antibiotics for different clonotypes as well as their minimal inhibitory concentrations, which also could differ by clonotype [36, 37].

Diagnostic reliability of clonal reference database is one of the most critical conditions to use it as a successful tool for guiding empiric treatment based on the clonal identify of the infecting agent. Indeed, compilation of such database went through multiple refining cycles [19, 20] to balance reference strains based on, among other parameters, patient demographics and clinical presentation. Furthermore, continued sustainability and improvement of the quality of clonal diagnostics will require the database to incorporate self-learning features to fine tune to both local and global dynamics of the antimicrobial resistance.

Our study has limitations. First, it was observational, leaving in question whether clinicians would act on guidance from the 7-SNP test, and the test's actual clinical impact. (Notably, the clinicians did not adhere to IDSA-recommended empirical regimens.) Second, it focused on *E*. *coli*-containing urine samples, whereas other organisms cause a variable proportion of UTIs, depending on the context [5, 6]. Third, it used antibiotic/pathogen mismatch as a surrogate for ineffective therapy, which, although documented previously for T/S and FQs [38, 39], was not directly studied here. It also has notable strengths, including its prospective cohort design, urgent care clinic setting, attention to point-of-care feasibility, local and multi-national reference databases, ample-sized study population, and assessment of test performance with respect to turn-around time and potential impact on prescribing.

As a key strategy for confronting the threat of antimicrobial resistance, the WHO 2014 report lists the need to “improve antimicrobial use supervision and support of clinical practices, especially diagnostic and treatment strategies” [40]. This study provides proof-of-principle that clonal diagnostics is a promising approach for optimizing empirical antibiotic therapy, promoting antimicrobial stewardship, and tracking the dynamics of emerging antibiotic-resistant pathogens.

## Supporting information

S1 Table Local reference set Table of clonotype-specific antibiograms compiled based on 1,225 *E*. *coli* urine isolates obtained from Kaiser Permanente Washington patients. (DOCX)

S2 Table Comparison of antibiotic resistance prevalence among study vs. local and multi-national (non-Seattle) reference *Escherichia coli* isolates for the 10 most frequent study clonotypes. (DOCX)

S1 Fig Correlation between *Escherichia coli* load in urine (by culture) and time-to-positive for the 7SNP-test. Data are for all test-positive samples (N = 274). Bubble size corresponds to the relative number of samples. According to linear regression the average time required for the test to detect *E*. *coli* is shortened by 1.8 min (95% CI 1.5–2.1, P < .001, R^2^ = .39) for every log_10_ increase in the urine *E*. *coli* concentration.
(TIFF)

S2 Fig Allowed trimethoprim/sulfamethoxazole (T/S) use at different thresholds and different reference sets. As the validation set we used the 220 subjects whose urine was positive for *E*. *coli* both by culture and 7-SNP test, and who had an antibiotic prescribed at the index visit or the next day. As the reference set we used either the local reference set (A) or a multinational reference set (B). For 10%, 20% and 30% resistance prevalence thresholds we calculated the percent of allowed T/S use (solid grey line) and the rate of mismatches in allowed cases (solid red line) and in disallowed cases (dotted red line).
(TIFF)

S1 Dataset *Escherichia coli* isolates from the local reference collection. Following variables are listed: ID (isolate’s name in UW collection), clonotype (septatype), resistance to seven antibiotics (AMP, ampicillin, CZ, cefazolin, CTR, ceftriaxone, T/S, trimethoprim/sulfamethoxazole, CIP, ciprofloxacin, NIT, nitrofurantoin, FOS, fosfomycin). Resistance is given in ‘1’ for resistant, ‘0’ for susceptible, ‘.’ for missing data.
(XLSX)

S2 Dataset *Escherichia coli* isolates from the multi-national (non-Seattle) reference collection. Following variables are listed: ID (isolate’s name in UW collection), clonotype (septatype), resistance to six antibiotics (AMP, ampicillin, CZ, cefazolin, CTR, ceftriaxone, T/S, trimethoprim/sulfamethoxazole, CIP, ciprofloxacin, NIT, nitrofurantoin). Resistance is given in ‘1’ for resistant, ‘0’ for susceptible, ‘.’ for missing data.
(XLSX)

S3 Dataset Samples from prospective clonal diagnostic study. Following variables are listed: study ID (name of the de-identified sample); bacterial load determined by 7-SNP test and bacterial load determined by culture (log10 cfu/ml); clonotype (septatype) determined in 7-SNP test directly from urine sample and clonotype from cultured bacteria; and resistance to seven antibiotics (AMP, ampicillin, CZ, cefazolin, CTR, ceftriaxone, T/S, trimethoprim/sulfamethoxazole, CIP, ciprofloxacin, NIT, nitrofurantoin, FOS, fosfomycin). Resistance is given in ‘1’ for resistant, ‘0’ for susceptible, ‘.’ for missing data.
(XLSX)

## References

[1] Johnson CC. Definitions, classification, and clinical presentation of urinary tract infections. Med Clin North Am. 1991;75(2):241–52. 1996031 10.1016/s0025-7125(16)30451-5

[2] Little P, Merriman R, Turner S, Rumsby K, Warner G, Lowes JA, et al Presentation, pattern, and natural course of severe symptoms, and role of antibiotics and antibiotic resistance among patients presenting with suspected uncomplicated urinary tract infection in primary care: observational study. BMJ. 2010;340:b5633 10.1136/bmj.b5633 20139213 PMC2817050

[3] Saint S. Clinical and economic consequences of nosocomial catheter-related bacteriuria. Am J Infect Control. 2000;28(1):68–75. 10679141 10.1016/s0196-6553(00)90015-4

[4] Wilson ML, Gaido L. Laboratory diagnosis of urinary tract infections in adult patients. Clin Infect Dis. 2004;38(8):1150–8. 10.1086/383029 15095222

[5] Flores-Mireles AL, Walker JN, Caparon M, Hultgren SJ. Urinary tract infections: epidemiology, mechanisms of infection and treatment options. Nat Rev Microbiol. 2015;13(5):269–84 10.1038/nrmicro3432 25853778 PMC4457377

[6] Foxman B. The epidemiology of urinary tract infection. Nat Rev Urol. 2010;7(12):653–60 10.1038/nrurol.2010.190 21139641

[7] Foxman B. Urinary tract infection syndromes: occurrence, recurrence, bacteriology, risk factors, and disease burden. Infect Dis Clin North Am. 2014;28(1):1–13. 10.1016/j.idc.2013.09.003 24484571

[8] Schappert SM, Rechtsteiner EA. Ambulatory medical care utilization estimates for 2007. Vital Health Stat 13 2011;(169):1–38. 21614897

[9] Gopal Rao G, Patel M. Urinary tract infection in hospitalized elderly patients in the United Kingdom: the importance of making an accurate diagnosis in the post broad-spectrum antibiotic era. J Antimicrob Chemother. 2009;63(1):5–6. 10.1093/jac/dkn458 19022779

[10] Falagas ME, Kotsantis IK, Vouloumanou EK, Rafailidis PI. Antibiotics versus placebo in the treatment of women with uncomplicated cystitis: a meta-analysis of randomized controlled trials. J Infect. 2009;58(2):91–102. 10.1016/j.jinf.2008.12.009 19195714

[11] Gupta K, Hooton TM, Naber KG, Wullt B, Colgan R, Miller LG, et al International clinical practice guidelines for the treatment of acute uncomplicated cystitis and pyelonephritis in women: A 2010 update by the Infectious Diseases Society of America and the European Society for Microbiology and Infectious Diseases. Clin Infect Dis. 2011;52(5):e103–20. 10.1093/cid/ciq257 21292654

[12] Caterino JM, Weed SG, Espinola JA, Camargo CA Jr. National trends in emergency department antibiotic prescribing for elders with urinary tract infection, 1996–2005. Acad Emerg Med. 2009;16(6):500–7. 10.1111/j.1553-2712.2009.00353.x 19245373

[13] Johnson L, Sabel A, Burman WJ, Everhart RM, Rome M, MacKenzie TD, et al Emergence of fluoroquinolone resistance in outpatient urinary Escherichia coli isolates. Am J Med. 2008;121(10):876–84. 10.1016/j.amjmed.2008.04.039 18823859

[14] Naber KG, Schito G, Botto H, Palou J, Mazzei T. Surveillance study in Europe and Brazil on clinical aspects and Antimicrobial Resistance Epidemiology in Females with Cystitis (ARESC): implications for empiric therapy. Eur Urol. 2008;54(5):1164–75. 10.1016/j.eururo.2008.05.010 18511178

[15] Taur Y, Smith MA. Adherence to the Infectious Diseases Society of America guidelines in the treatment of uncomplicated urinary tract infection. Clin Infect Dis. 2007;44(6):769–74. 10.1086/511866 17304445

[16] Ali MH, Kalima P, Maxwell SR. Failure to implement hospital antimicrobial prescribing guidelines: a comparison of two UK academic centres. J Antimicrob Chemother. 2006;57(5):959–62. 10.1093/jac/dkl076 16531431

[17] Hecker MT, Fox CJ, Son AH, Cydulka RK, Siff JE, Emerman CL, et al Effect of a stewardship intervention on adherence to uncomplicated cystitis and pyelonephritis guidelines in an emergency department setting. PLoS One. 2014;9(2):e87899 10.1371/journal.pone.0087899 24498394 PMC3912125

[18] Grigoryan L, Zoorob R, Wang H, Trautner BW. Low Concordance With Guidelines for Treatment of Acute Cystitis in Primary Care. Open Forum Infect Dis. 2015;2(4):ofv159 10.1093/ofid/ofv159 26753168 PMC4675917

[19] Tchesnokova V, Avagyan H, Billig M, Chattopadhyay S, Aprikian P, Chan D, et al A Novel 7-Single Nucleotide Polymorphism-Based Clonotyping Test Allows Rapid Prediction of Antimicrobial Susceptibility of Extraintestinal Directly From Urine Specimens. Open Forum Infect Dis. 2016;3(1):ofw002 10.1093/ofid/ofw002 26925427 PMC4766386

[20] Tchesnokova V, Billig M, Chattopadhyay S, Linardopoulou E, Aprikian P, Roberts PL, et al Predictive diagnostics for Escherichia coli infections based on the clonal association of antimicrobial resistance and clinical outcome. J Clin Microbiol. 2013;51(9):2991–9. 10.1128/JCM.00984-13 23843485 PMC3754640

[21] Price LB, Johnson JR, Aziz M, Clabots C, Johnston B, Tchesnokova V, et al The epidemic of extended-spectrum-beta-lactamase-producing Escherichia coli ST131 is driven by a single highly pathogenic subclone, H30-Rx. MBio. 2013;4(6):e00377–13. 10.1128/mBio.00377-13 24345742 PMC3870262

[22] Fridkin SK, Hill HA, Volkova NV, Edwards JR, Lawton RM, Gaynes RP, et al Temporal changes in prevalence of antimicrobial resistance in 23 US hospitals. Emerg Infect Dis. 2002;8(7):697–701 10.3201/eid0807.010427 12095437 PMC2730337

[23] Guyomard-Rabenirina S, Malespine J, Ducat C, Sadikalay S, Falord M, Harrois D, et al Temporal trends and risks factors for antimicrobial resistant Enterobacteriaceae urinary isolates from outpatients in Guadeloupe. BMC Microbiol. 2016;16(1):121 10.1186/s12866-016-0749-9 27342199 PMC4919840

[24] Swami SK, Banerjee R. Comparison of hospital-wide and age and location—stratified antibiograms of S. aureus, E. coli, and S. pneumoniae: age- and location-stratified antibiograms. Springerplus. 2013;2(1):63 10.1186/2193-1801-2-63 23487499 PMC3593003

[25] CLSI. Performance Standards for Antimicrobial Susceptibility Testing; Twentieth Information Supplement. 2010.

[26] Hooton TM, Roberts PL, Cox ME, Stapleton AE. Voided midstream urine culture and acute cystitis in premenopausal women. N Engl J Med. 2013;369(20):1883–91. 10.1056/NEJMoa1302186 24224622 PMC4041367

[27] Shin J, Kim J, Wie SH, Cho YK, Lim SK, Shin SY, et al Fluoroquinolone resistance in uncomplicated acute pyelonephritis: epidemiology and clinical impact. Microb Drug Resist. 2012;18(2):169–75. 10.1089/mdr.2011.0139 22400491

[28] Johnson JR, Thuras P, Johnston BD, Weissman SJ, Limaye AP, Riddell K, et al The Pandemic H30 Subclone of Escherichia coli Sequence Type 131 Is Associated With Persistent Infections and Adverse Outcomes Independent From Its Multidrug Resistance and Associations With Compromised Hosts. Clin Infect Dis. 2016;62(12):1529–36. 10.1093/cid/ciw193 27025834 PMC4885656

[29] Talan DA, Stamm WE, Hooton TM, Moran GJ, Burke T, Iravani A, et al Comparison of ciprofloxacin (7 days) and trimethoprim-sulfamethoxazole (14 days) for acute uncomplicated pyelonephritis pyelonephritis in women: a randomized trial. JAMA. 2000;283(12):1583–90. 10735395 10.1001/jama.283.12.1583

[30] Caliendo AM, Gilbert DN, Ginocchio CC, Hanson KE, May L, Quinn TC, et al Better tests, better care: improved diagnostics for infectious diseases. Clin Infect Dis. 2013;57 Suppl 3:S139–70. 24200831 10.1093/cid/cit578 PMC3820169

[31] ECDC. Transatlantic Task Force on Antimicrobial Resistance Report on the Joint EU-US Workshop: challenges and solutions in the development of new diagnostic tests to combat antimicrobial resistance. 2011.

[32] Brolund A, Sundqvist M, Kahlmeter G, Grape M. Molecular characterisation of trimethoprim resistance in Escherichia coli and Klebsiella pneumoniae during a two year intervention on trimethoprim use. PLoS One. 2010;5(2):e9233 10.1371/journal.pone.0009233 20169085 PMC2821933

[33] Shin HW, Lim J, Kim S, Kim J, Kwon GC, Koo SH. Characterization of trimethoprim-sulfamethoxazole resistance genes and their relatedness to class 1 integron and insertion sequence common region in gram-negative bacilli. J Microbiol Biotechnol. 2015;25(1):137–42. 25348695 10.4014/jmb.1409.09041

[34] Redgrave LS, Sutton SB, Webber MA, Piddock LJ. Fluoroquinolone resistance: mechanisms, impact on bacteria, and role in evolutionary success. Trends Microbiol. 2014;22(8):438–45. 10.1016/j.tim.2014.04.007 24842194

[35] Hooper DC. Emerging mechanisms of fluoroquinolone resistance. Emerg Infect Dis. 2001;7(2):337–41. 10.3201/eid0702.700337 11294736 PMC2631735

[36] Johnson JR, Porter SB, Thuras P, Johnson TJ, Price LB, Tchesnokova V, et al Greater ciprofloxacin tolerance as a possible selectable phenotype underlying the pandemic spread of the H30 subclone of Escherichia coli sequence type 131. Antimicrob Agents Chemother. 2015;59(11):7132–5. 10.1128/AAC.01687-15 26324269 PMC4604381

[37] Johnson JR, Johnston B, Kuskowski MA, Sokurenko EV, Tchesnokova V. Intensity and Mechanisms of Fluoroquinolone Resistance within the H30 and H30Rx Subclones of Escherichia coli Sequence Type 131 Compared with Other Fluoroquinolone-Resistant E. coli. Antimicrob Agents Chemother. 2015;59(8):4471–80. 10.1128/AAC.00673-15 25987621 PMC4505254

[38] Lawrenson RA, Logie JW. Antibiotic failure in the treatment of urinary tract infections in young women. J Antimicrob Chemother. 2001;48(6):895–901. 11733475 10.1093/jac/48.6.895

[39] Gupta K, Stamm WE. Outcomes associated with trimethoprim/sulphamethoxazole (TMP/SMX) therapy in TMP/SMX resistant community-acquired UTI. Int J Antimicrob Agents. 2002;19(6):554–6. 12135847 10.1016/s0924-8579(02)00104-8

[40] WHO. WHO Global Strategy for Containment of Antimicrobial Resistance. World Health Organization, 2001.

