# Supplementary material for: Bacterial clonal diagnostics as a tool for evidence-based empiric antibiotic selection
Source: PLoS One. 2017 Mar 28;12(3):e0174132. doi: 10.1371/journal.pone.0174132 (PMC5369764; doi:10.1371/journal.pone.0174132)
Supplement: S1 Table — (DOCX) [file pone.0174132.s001.docx]

**S1 Table. Local reference set table of clonotype-specific antibiograms compiled based on 1,225 *E. coli* urine isolates obtained from Group Health patients.**

| **Clonotype** | Corresponding MLST Sequence Type(s) ^a^ | **Observed frequencies (%)** | **Resistance prevalence (%) ^c^** | | | | | | | |
| --- | --- | --- | --- | --- | --- | --- | --- | --- | --- | --- |
|  |  |  | **AMP** | **A/C** | **CZ** | **CTR** | **T/S** | **CIP** | **NIT** | **FOS** |
| ***E. coli*** | | 1225 (100) | 45 | 15 | 15 | 4 | 21 | 14 | 5 | 2 |
| **CT620** | ST73 (H9/H10) | 119 (9.7) | 48 | 23 | 20 | 1 | 15 | 4 | 5 | 1 |
| **CT530** | ST127 (H2) | 113 (9.2) | 27 | 7 | 5 | 0 | 5 | 1 | 4 | 1 |
| **CT271** | ST69 (H27) | 97 (7.9) | 75 | 19 | 20 | 5 | 56 | 14 | 8 | 3 |
| **CT760** | ST95 (H41) | 95 (7.8) | 23 | 4 | 4 | 0 | 6 | 4 | 3 | 0 |
| **CT561** | ST131 (H30) | 91 (7.4) | 75 | 31 | 38 | 13 | 45 | 86 | 10 | 4 |
| **CT361** | ST58 / ST88 (H31), ST354 / ST648 (H58) | 60 (4.9) | 38 | 12 | 13 | 7 | 17 | 8 | 8 | 4 |
| **CT571** | ST14 (H27/H64) ^b^ | 51 (4.2) | 73 | 12 | 10 | 0 | 24 | 25 | 4 | 9 |
| **CT371** | ST58 / ST648 / ST88 / ST405 (H27) | 46 (3.8) | 57 | 33 | 22 | 11 | 43 | 30 | 11 | 0 |
| **CT721** | ST95 (H15) | 45 (3.7) | 22 | 4 | 4 | 0 | 7 | 2 | 4 | 3 |
| **CT360** | ST58 (H61), ST88/101 (H86), ST61 (H44) | 40 (3.3) | 48 | 13 | 15 | 3 | 18 | 15 | 0 | 0 |
| **CT771** | ST10 (H23/27) / ST95 (H27) | 38 (3.1) | 47 | 13 | 13 | 5 | 39 | 11 | 5 | 0 |
| **CT531** | ST141 / ST491 (H5) | 37 (3.0) | 16 | 0 | 3 | 0 | 0 | 0 | 3 | 0 |
| **CT731** | ST569 (H5) | 36 (2.9) | 25 | 0 | 3 | 0 | 3 | 3 | 8 | 0 |
| **CT131** | ST12 (H5) | 32 (2.6) | 53 | 47 | 44 | 3 | 3 | 3 | 0 | 0 |
| **CT511** | ST1876 / ST429 (H20) | 32 (2.6) | 31 | 16 | 13 | 0 | 6 | 0 | 0 | 0 |
| **CT661** | ST73 (H30) | 31 (2.5) | 32 | 19 | 16 | 0 | 3 | 0 | 3 | 0 |
| **CT510** | ST131 (H22) | 26 (2.1) | 50 | 31 | 38 | 4 | 15 | 0 | 0 | 0 |
| **CT560** | ST131 (H41) | 26 (2.1) | 77 | 12 | 4 | 4 | 38 | 12 | 4 | 0 |
| **CT351** | ST69 (H54) / ST58 (H54) | 23 (1.9) | 39 | 13 | 13 | 9 | 26 | 17 | 4 | 0 |
| **CT761** | ST10 (H30/31) / ST95 (H30) | 23 (1.9) | 39 | 13 | 4 | 0 | 22 | 4 | 0 | 0 |

**S1 Table (continued).**

| **Clonotype** | Corresponding MLST Sequence Type(s) ^a^ | **Observed frequencies (%)** | **Resistance prevalence (%) ^c^** | | | | | | | |
| --- | --- | --- | --- | --- | --- | --- | --- | --- | --- | --- |
|  |  |  | **AMP** | **A/C** | **CZ** | **CTR** | **T/S** | **CIP** | **NIT** | **FOS** |
| **CT261** | ST69 / ST394 (H47) | 17 (1.4) | 41 | 0 | 12 | 0 | 18 | 0 | 18 | 0 |
| **CT751** | ST10 (H54) | 17 (1.4) | 29 | 18 | 18 | 12 | 29 | 29 | 0 | 9 |
| **CT520** | ST373 (H9/12) | 16 (1.3) | 38 | 13 | 13 | 6 | 19 | 0 | 0 | 0 |
| **CT260** | ST38 / ST59 (H65/H41) | 14 (1.1) | 50 | 14 | 7 | 7 | 21 | 14 | 7 | 0 |
| **CT231** | ST38 (H5) | 12 (1.0) | 75 | 25 | 25 | 8 | 58 | 17 | 0 | 0 |
| **CT630** | ST80 (H1) / ST73 (H2/154) | 12 (1.0) | 17 | 8 | 17 | 0 | 0 | 0 | 0 | 0 |
| **CT570** | ST117 (H97) | 11 (0.9) | 45 | 9 | 27 | 18 | 55 | 0 | 0 | 0 |
| **CT171** | ST12 (H27) | 9 (0.7) | 22 | 11 | 11 | 0 | 11 | 11 | 11 | 0 |
| **CT350** | ST405 (H56) | 6 (0.5) | 83 | 50 | 50 | 0 | 83 | 0 | 0 | 0 |
| **CT711** | ST676 (H21) / ST2551 (H20) | 6 (0.5) | 0 | 0 | 0 | 0 | 0 | 0 | 0 | 0 |
| **CT500** | ST636 (H0) | 5 (0.4) | 40 | 20 | 0 | 0 | 60 | 0 | 0 | 0 |
| **CT551** | ST144 (H54) / ST2086 (H57) | 5 (0.4) | 20 | 0 | 0 | 0 | 0 | 0 | 20 | 0 |
| **CT331** | ST648 (H5) / ST38 (H5) | 4 (0.3) | 50 | 0 | 0 | 0 | 25 | 50 | 0 | 0 |
| **CT300** | ST2141 (H0) / ST2711 (H0) | 3 (0.2) | 67 | 33 | 33 | 33 | 33 | 0 | 0 | 0 |
| **CT330** | ST58 (H2) / ST2250 (H2) | 3 (0.2) | 0 | 0 | 0 | 0 | 0 | 0 | 0 | 0 |
| **CT730** | ST73 (H154) / ST1444 (H3) | 3 (0.2) | 0 | 0 | 0 | 0 | 0 | 0 | 0 | 0 |
| **CT160** | ST12 (H41) | 2 (0.2) | 50 | 0 | 0 | 0 | 0 | 0 | 0 | 0 |
| **CT370** | ST991 / ST1882 (H123) | 2 (0.2) | 0 | 0 | 50 | 0 | 0 | 0 | 0 | 0 |
| **CT550** | ST2015 (H197) | 2 (0.2) | 0 | 0 | 0 | 0 | 0 | 0 | 0 | 0 |

**S1 Table (continued).**

| **Clonotype** | Corresponding MLST Sequence Type(s) ^a^ | **Observed frequencies (%)** | **Resistance prevalence (%) ^c^** | | | | | | | |
| --- | --- | --- | --- | --- | --- | --- | --- | --- | --- | --- |
|  |  |  | **AMP** | **A/C** | **CZ** | **CTR** | **T/S** | **CIP** | **NIT** | **FOS** |
| **CT120** | ST12 (H9) | 1 (0.1) | 0 | 0 | 0 | 0 | 100 | 0 | 0 | 0 |
| **CT211** | ST69 (H21) | 1 (0.1) | 0 | 0 | 0 | 0 | 0 | 0 | 0 | 0 |
| **CT420** | ST73 (H9) | 1 (0.1) | 100 | 0 | 100 | 0 | 100 | 0 | 0 | 0 |
| **CT621** | ST73 (H15) | 1 (0.1) | 0 | 0 | 0 | 0 | 0 | 0 | 0 | 0 |
| **CT671** | ST73 (H27) / ST706 (H25) | 1 (0.1) | 0 | 0 | 0 | 0 | 0 | 0 | 0 | 0 |
| **CT100** | ST12 (H0) | 1 (0.1) | 100 | 100 | 100 | 0 | 0 | 0 | 0 | 100 |
| **CT320** | ST10309 (H9) | 1 (0.1) | 0 | 0 | 0 | 0 | 0 | 100 | 0 | 0 |
| **CT461** | ST73 (H30) | 1 (0.1) | 100 | 100 | 100 | 0 | 0 | 0 | 0 | 0 |
| **CT521** | ST636 (H15) | 1 (0.1) | 0 | 0 | 0 | 0 | 0 | 0 | 0 | 0 |
| **CT600** | ST73 (H0) | 1 (0.1) | 0 | 0 | 0 | 0 | 0 | 0 | 0 | 0 |
| **CT651** | ST706 (H229) / ST73 (H54) | 1 (0.1) | 100 | 0 | 0 | 0 | 0 | 0 | 0 | 0 |
| **CT660** | ST73 (H41) | 1 (0.1) | 0 | 0 | 0 | 0 | 0 | 0 | 0 | 0 |
| **CT700** | ST10 (H0) | 1 (0.1) | 0 | 0 | 0 | 0 | 0 | 0 | 0 | 0 |
| **CT710** | ST803 (H88) | 1 (0.1) | 0 | 0 | 0 | 0 | 0 | 0 | 0 | 100 |
| **CT720** | ST2622 (H75) / ST2474 (H9) | 1 (0.1) | 0 | 0 | 0 | 0 | 0 | 0 | 0 | 0 |

^a^ For each CT (clonotype) the most prevalent (≥ 90% of isolates) sequence type (ST), as determined by multilocus sequence typing, is shown, with its subclone determined by CH typing (H) in parentheses, where applicable. For CTs represented by multiple STs, each accounting for ≤ 90% of the constituent isolates, up to four major STs and corresponding H subclones are listed

^b^ CT571 comprises ST14, ST404 and ST1193 which belong to clonal complex of ST14

^c^ For local reference set antibiotic resistance prevalence for all *E. coli* and for every clonotype is given as percent resistant isolates from total number of isolates, overall or within clonotype; green color indicates resistance below conventional threshold (≤20%), red color – above it (>20%).
