# Supplementary material for: Bacterial clonal diagnostics as a tool for evidence-based empiric antibiotic selection
Source: PLoS One. 2017 Mar 28;12(3):e0174132. doi: 10.1371/journal.pone.0174132 (PMC5369764; doi:10.1371/journal.pone.0174132)
Supplement: S2 Table — (DOCX) [file pone.0174132.s002.docx]

**S2 Table. Local reference set table of clonotype-specific antibiograms compiled based on 1,225 *E. coli* urine isolates obtained from Group Health patients.**

| **Clono-type** | **Corresponding MLST Sequence Type(s)** ^a^ | **no. isolates** | | | **Resistance prevalence (%) ^c^** | | | | | | | | | | | | | | | | | |
| --- | --- | --- | --- | --- | --- | --- | --- | --- | --- | --- | --- | --- | --- | --- | --- | --- | --- | --- | --- | --- | --- | --- |
|  |  |  |  |  | **AMP** | | | **CZ** | | | **CTR** | | | **T/S** | | | **CIP** | | | **NIT** | | |
|  |  | **Study** | **Local** | **Non-Seattle** | **Study** | **Local** | **Non-Seattle** | **Study** | **Local** | **Non-Seattle** | **Study** | **Local** | **Non-Seattle** | **Study** | **Local** | **Non-Seattle** | **Study** | **Local** | **Non-Seattle** | **Study** | **Local** | **Non-Seattle** |
| **All *E. coli*** | | 267 | 1225 | 741 | 48 | 45 | 61* | 8 | 15* | 31* | 5 | 4 | 17* | 25 | 21 | 37* | 21 | 14* | 32* | 0.4 | 5* | 11* |
| **CT561** | **ST131 (H30)** | 40 | 91 | 75 | 78 | 75 | 95* | 28 | 38 | 62* | 15 | 13 | 41* | 38 | 45 | 43 | 85 | 86 | 95 | 3 | 10 | 5 |
| **CT620** | **ST73 (H9/H10)** | 23 | 119 | 57 | 30 | 48 | 40 | 0 | 20* | 18* | 0 | 1 | 4 | 4 | 15 | 12 | 0 | 4 | 2 | 0 | 5 | 9 |
| **CT760** | **ST95 (H41)** | 22 | 95 | 16 | 41 | 23 | 19 | 0 | 4 | 6 | 0 | 0 | 6 | 0 | 6 | 6 | 0 | 4 | 6 | 0 | 3 | 0 |
| **CT530** | **ST127 (H2)** | 19 | 113 | 49 | 21 | 27 | 35 | 5 | 5 | 4 | 0 | 0 | 0 | 5 | 5 | 16 | 0 | 1 | 0 | 0 | 4 | 4 |
| **CT271** | **ST69 (H27)** | 17 | 97 | 41 | 94 | 75 | 73 | 12 | 20 | 27 | 6 | 5 | 12 | 82 | 56 | 56 | 12 | 14 | 24 | 0 | 8 | 15 |
| **CT571** | **ST14 (H27/H64)** ^b^ | 14 | 51 | 16 | 71 | 73 | 44 | 0 | 10 | 19 | 0 | 0 | 6 | 36 | 24 | 19 | 57 | 25* | 25 | 0 | 4 | 6 |
| **CT531** | **ST141 (H5), ST491 (H5)** | 13 | 37 | 26 | 38 | 16 | 38 | 0 | 3 | 15 | 0 | 0 | 8 | 8 | 0 | 12 | 0 | 0 | 0 | 0 | 3 | 12 |
| **CT661** | **ST73 (H30)** | 12 | 31 | 17 | 25 | 32 | 29 | 0 | 16 | 12 | 0 | 0 | 0 | 0 | 3 | 18 | 0 | 0 | 0 | 0 | 3 | 6 |
| **CT721** | **ST95 (H15)** | 12 | 45 | 22 | 10 | 32 | 36 | 0 | 7 | 14 | 0 | 0 | 5 | 0 | 9 | 14 | 0 | 3 | 18 | 0 | 6 | 9 |
| **CT361** | **ST58 (H31), ST88 (H31), ST354 (H58), ST648 (H58)** | 10 | 60 | 83 | 30 | 38 | 75* | 0 | 13 | 39* | 0 | 7 | 17 | 30 | 17 | 54 | 0 | 8 | 57* | 0 | 8 | 19 |

^a^ For each CT (clonotype) the most prevalent (≥ 90% of isolates) sequence type (ST), as determined by multilocus sequence typing, is shown, with its subclone determined by CH typing (H) in parentheses, where applicable. For CTs represented by multiple STs, each accounting for ≤ 90% of the constituent isolates, up to four major STs and corresponding H subclones are listed

^b^ CT571 comprises ST14, ST404 and ST1193 which belong to clonal complex of ST14

^c^ For study set, local reference set (‘Local’) and multi-national reference set (non-Seattle) antibiotic resistance prevalence for all *E. coli* and for major clonotypes is given as percent resistant isolates from total number of isolates, overall or within clonotype; the level within study and reference sets was compared individually for each antibiotic using two-sided Fisher’s exact test; * P < .05; data for fosfomycin for multi-national reference set are not available.
