# Supplementary figures and images for: Bacterial clonal diagnostics as a tool for evidence-based empiric antibiotic selection
Source: PLoS One. 2017 Mar 28;12(3):e0174132. doi: 10.1371/journal.pone.0174132 (PMC5369764; doi:10.1371/journal.pone.0174132)

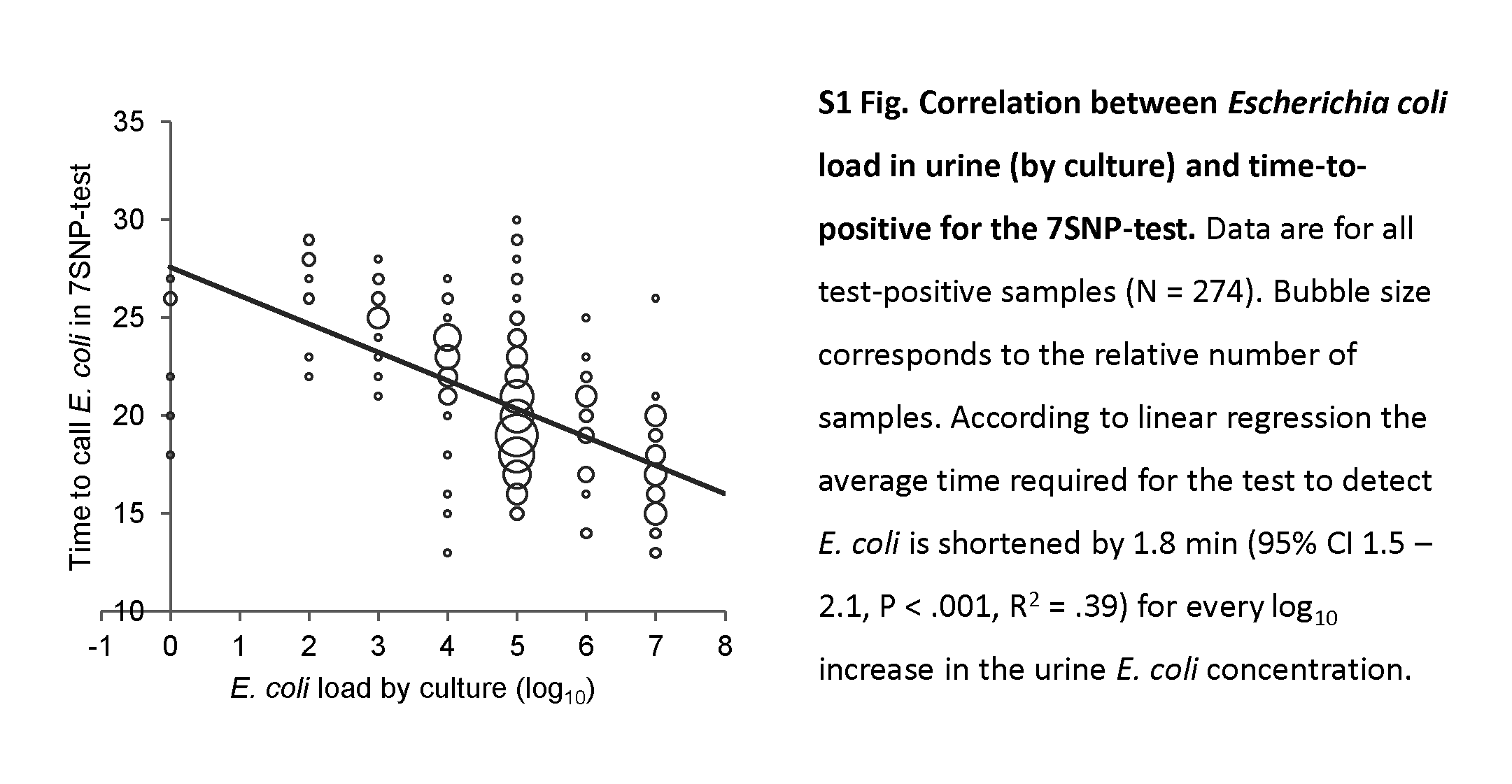

Supplement: S1 Fig — Data are for all test-positive samples (N = 274). Bubble size corresponds to the relative number of samples. According to linear regression the average time required for the test to detect E. coli is shortened by 1.8 min (95% CI 1.5–2.1, P < .001, R2 = .39) for every log10 increase in the urine E. coli concentration. (TIFF) [file pone.0174132.s003.tiff]

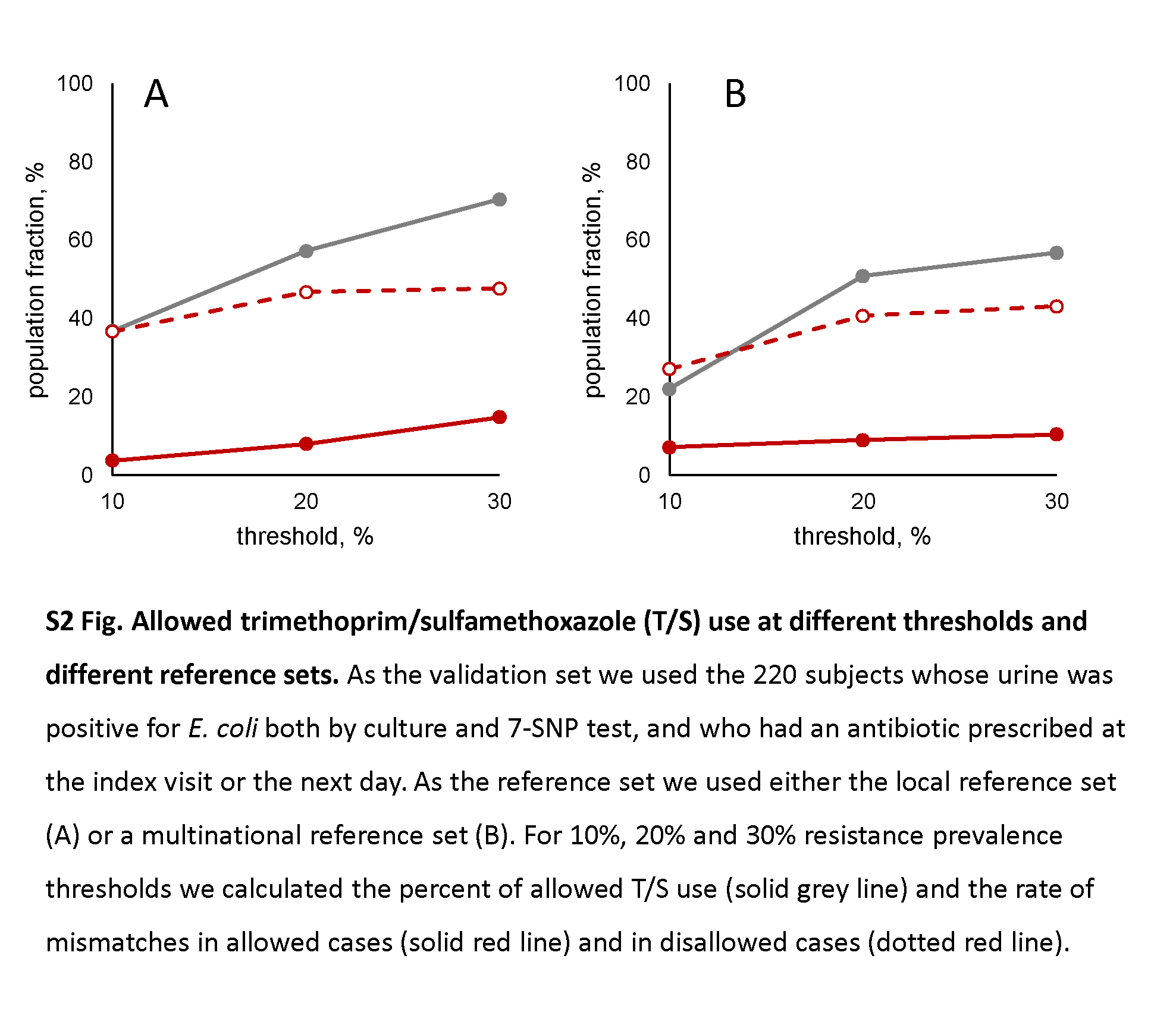

Supplement: S2 Fig — As the validation set we used the 220 subjects whose urine was positive for E. coli both by culture and 7-SNP test, and who had an antibiotic prescribed at the index visit or the next day. As the reference set we used either the local reference set (A) or a multinational reference set (B). For 10%, 20% and 30% resistance prevalence thresholds we calculated the percent of allowed T/S use (solid grey line) and the rate of mismatches in allowed cases (solid red line) and in disallowed cases (dotted red line). (TIFF) [file pone.0174132.s004.tiff]
